# Anti-HER2 antibody enhances the growth inhibitory effect of anti-oestrogen on breast cancer cells expressing both oestrogen receptors and HER2

**DOI:** 10.1054/bjoc.1999.0875

**Published:** 1999-12-08

**Authors:** H Kunisue, J Kurebayashi, T Otsuki, C K Tang, M Kurosumi, S Yamamoto, K Tanaka, H Doihara, N Shimizu, H Sonoo

**Affiliations:** Department of Breast and Thyroid Surgery, Department of Hygiene, Kawasaki Medical School, 577 Matsushima Kurashiki, Okayama, 701-0192, Japan; Lombardi Cancer Center, Georgetown Medical Center, Washington, DC20007, USA; Department of Pathology, Saitama Cancer Center, Kitaadachi-gun, Saitama, 362, Japan; Department of Surgery II, Okayama University School of Medicine, Okayama, Okayama700-8558, Japan

**Keywords:** anti-HER2 antibody, anti-oestrogen, breast cancer, additive effect, apoptosis

## Abstract

Anti-oestrogen is effective for the treatment of oestrogen receptor (ER)-positive breast carcinomas, butmost of these tumours become resistant to anti-oestrogen. It has been suggested that anti-oestrogen therapy may induce a HER2signalling pathway in breast cancer cells and this may cause resistance to anti-oestrogen. Thus, it is conceivable that combinedtherapy with anti-oestrogen and anti-HER2 antibody might be more effective. In the present study, we investigated the effect ofcombined treatment with a humanized anti-HER2 monoclonal antibody, rhumAbHER2 (trastuzumab), and an anti-oestrogen, ICI 182,780, onthe cell growth of three human breast cancer cell lines which respectively express different levels of ER and HER2. The combinedtreatment enhanced the growth inhibitory effect on ML-20 cells, which express a high level of ER and a moderate level of HER2, butshowed no additive effect on either KPL-4 cells, which express no ER and a moderate level of HER2, or MDA-MB-231 cells, whichexpress no ER and a low level of HER2. It is also suggested that both the antibody and anti-oestrogen induce a G1–S blockadeand apoptosis. These findings indicate that combined treatment with anti-HER2 antibody and anti-oestrogen may be useful for thetreatment of patients with breast cancer expressing both ER and HER2. © 2000 Cancer Research Campaign


*British Journal of Cancer* (2000) **82**(1), 46–51
© 2000 Cancer Research Campaign
Article no. bjoc.1999.0875

### Anti-HER2 antibody enhances the growth inhibitory effect of anti-oestrogen on breast cancer cells expressing both oestrogen receptors and HER2

**H Kunisue[1,5], J Kurebayashi[1], T Otsuki[2], CK Tang[3], M Kurosumi[4], S Yamamoto[1], K Tanaka[1], H Doihara[5], N Shimizu[5] and H Sonoo[1]**

[1]Department of Breast and Thyroid Surgery, [2]Department of Hygiene, Kawasaki Medical School, 577 Matsushima Kurashiki, Okayama 701-0192, Japan; [3]Lombardi Cancer Center, Georgetown Medical Center, Washington, DC 20007, USA; [4]Department of Pathology, Saitama Cancer Center, Kitaadachi-gun, Saitama 362, Japan; [5]Department of Surgery II, Okayama University School of Medicine, Okayama, Okayama 700-8558, Japan

**Summary** Anti-oestrogen is effective for the treatment of oestrogen receptor (ER)-positive breast carcinomas, but most of these tumours become resistant to anti-oestrogen. It has been suggested that anti-oestrogen therapy may induce a HER2 signalling pathway in breast cancer cells and this may cause resistance to anti-oestrogen. Thus, it is conceivable that combined therapy with anti-oestrogen and anti-HER2 antibody might be more effective. In the present study, we investigated the effect of combined treatment with a humanized anti-HER2 monoclonal antibody, rhumAbHER2 (trastuzumab), and an anti-oestrogen, ICI 182,780, on the cell growth of three human breast cancer cell lines which respectively express different levels of ER and HER2. The combined treatment enhanced the growth inhibitory effect on ML-20 cells, which express a high level of ER and a moderate level of HER2, but showed no additive effect on either KPL-4 cells, which express no ER and a moderate level of HER2, or MDA-MB-231 cells, which express no ER and a low level of HER2. It is also suggested that both the antibody and anti-oestrogen induce a G1–S blockade and apoptosis. These findings indicate that combined treatment with anti-HER2 antibody and anti-oestrogen may be useful for the treatment of patients with breast cancer expressing both ER and HER2. © 2000 Cancer Research Campaign

**Keywords**: anti-HER2 antibody; anti-oestrogen; breast cancer; additive effect; apoptosis

*HER2/c-erb B-2* is the homologue of the rat proto-oncogene *neu* (Schecter et al, 1984) and is located on chromosome 17q21 (Coussens et al, 1985; Fukushige et al, 1986). It encodes a 185-kDa transmembrane glycoprotein receptor (p185HER2) that has intrinsic tyrosine kinase activity (Maguire et al, 1989). An anti-HER2 monoclonal antibody, rhumAbHER2 (trastuzumab), is a humanized form of the murine 4D5 antibody which is directed to the external domain of HER2 and inhibits the growth of cells overexpressing HER2 (Carter et al, 1992; Tokuda et al, 1996; Baselga et al, 1998). Recently, clinical phase studies with rhumAbHER2 as a single agent were conducted in patients with metastatic breast cancer overexpressing HER2, and 12 and 15% response rates were reported (Baselga et al, 1996; Cobleigh et al, 1998). In addition, two clinical studies with chemotherapy combined with rhumAbHER2 were conducted and demonstrated that the addition of rhumAbHER2 to chemotherapy significantly increased not only the response rate but also the duration of response (Pegram et al, 1998; Slamon et al, 1998). Very recently, this antibody has been approved for clinical use with patients having HER2-overexpressing breast cancer in the United States.

It has been reported that either amplification of the *HER2* proto-oncogene or overexpression of HER2 has been observed in 25–30% of primary breast cancers and has correlated with a poor prognosis (Slamon et al, 1987; Wright et al, 1989). Approximately half of these cancers were oestrogen receptor (ER)-positive (McCann et al, 1991). It has been also suggested that, in the adjuvant setting, an anti-oestrogen, tamoxifen, may worsen disease-free survival of patients with breast cancer expressing both ER and HER2 (Bianco et al, 1998).

Anti-oestrogens are well known to be effective for the treatment of ER-positive breast carcinomas. Unfortunately, however, most of them eventually develop resistance to anti-oestrogens. It is also known that anti-oestrogens are less effective for the treatment of patients with breast cancer overexpressing HER1 or HER2 together with ER (Ross and Fletcher, 1998). Previous experiments have indicated that oestrogen down-regulates HER2 in ER-positive breast cancer cells and anti-oestrogen reverses the oestrogen-induced decrease in HER2 expression (Read et al, 1990). Thus, it is conceivable that anti-oestrogen may activate a HER2-signalling pathway and may cause resistance to anti-oestrogen. If so, it seems reasonable to use both anti-oestrogen and anti-HER2 antibody for the treatment of patients with breast cancer expressing both ER and HER2.

In the present study, we investigated the effects of combined treatment with a steroidal, pure anti-oestrogen, ICI 182,780, and a humanized anti-HER2 monoclonal antibody, rhumAbHER2, in three breast cancer cell lines which respectively express different levels of ER and HER2 to explore the possible additive effect of the combined treatment.

*Received 12 April 1999*
*Revised 21 June 1999*
*Accepted 22 June 1999*

*Correspondence to:* J Kurebayashi

### MATERIALS AND METHODS

### Human breast cancer cell lines

The ML-20 cell line is a transfectant of the MCF-7 cell line with the pCHC β-Gal expression vector encoding the bacterial *lacZ* gene (Kurebayashi et al, 1993). The MDA-MB-231 cell line was kindly provided by Dr Robert B Dickson (Lombardi Cancer Center, Georgetown University Medical Center, Washington, DC, USA). The KPL-4 cell line was established in our laboratory and its characterization has been published elsewhere (Kurebayashi et al, 1999). This cell line was derived from the malignant pleural effusion of a Japanese patient with recurrent breast cancer. All of the cell lines were routinely cultured in Dulbecco's modified Eagle's medium (DMEM; ICN Biochemicals, Costa Mesa, CA, USA) supplemented with 5% fetal bovine serum (FBS; ICN Biochemicals Japan, Osaka, Japan).

### Reagents

The steroidal, pure anti-oestrogen, ICI 182,780, was kindly provided by Zeneca Pharmaceuticals (Macclesfield, UK). ICI 182,780 was dissolved with 100% ethanol and added to the medium at a final ethanol concentration of 0.1%. The humanized anti-ErbB-2 monoclonal antibody, rhumAbHER2, was kindly provided by Mitsubishi Chemical Co. (Tokyo, Japan).

### ER analysis

Oestrogen receptor (ER) levels in the cell pellet of the three cell lines were measured by an enzyme immunoassay using ER-EIA kits (Dinabot, Tokyo, Japan) following the manufacturer's recommendation.

### Flow cytometric analysis of HER family member expression

Approximately $1 \times 10^6$ cells per sample were harvested with trypsin, stained with first antibodies for 1 h and washed with phosphate-buffered saline (PBS) twice. Then they were stained with secondary fluoroscein isothiocyanate (FITC)-antimouse antibody (Becton Dickinson, San Jose, CA, USA) for 30 min and washed with PBS twice. The level of each HER family member was analysed by a flow cytometer (Becton Dickinson). The first antibodies were: anti-HER1 monoclonal antibody (Oncogene Science, Uniondale, NY, USA), anti-HER2 monoclonal antibody (Ab-2, NeoMarker, Freemont, CA, USA), anti-HER3 monoclonal antibody (NeoMarker) and anti-HER4 monoclonal antibody (NeoMarker). The level of each HER family member was expressed as the product of the specific peak fluorescence intensity divided by the background peak intensity (Kurebayashi et al, 1999).

### Cell growth in vitro

Three cell lines were respectively plated at a density of $5 \times 10^4$ cells per well in 12-well plates (SB Medical, Tokyo, Japan) and grown in DMEM supplemented with 5% fetal bovine serum (FBS) at 37°C in a 5% carbon dioxide atmosphere for 2 days. The cells were then washed with PBS and incubated with phenol red-free RPMI-1640 medium (Gibco-BRL, Bethesda, MD, USA) supplemented with 2% dextran-coated charcoal-stripped FBS (Kurebayashi et al, 1998) plus $10^{-9}$–$10^{-7}$ M ICI 182,780, 0.1–10 μg ml$^{-1}$ rhumAbHER2 or $10^{-7}$ M ICI 182,780 and/or 10 μg ml$^{-1}$ of rhumAbHER2. The culture medium was changed every other day. Triplicate wells were trypsinized 4 days after switching the culture medium and the cell number was counted with a Coulter counter (Coulter Electronics, Harpenden, UK).

### Cell cycle analysis

Three cell lines were plated at a density of $5 \times 10^4$ cells per well in 12-well plates and grown in DMEM supplemented with 5% FBS for 3 days. Then the cells were washed with PBS and treated with phenol red-free RPMI-1640 medium supplemented with 2% dextran-coated charcoal-stripped FBS plus $10^{-7}$ M ICI 182,780, 10 μg ml$^{-1}$ rhumAbHER2 or both. Triplicate wells were trypsinized, harvested a day after and stained with propidium iodide using the CycleTest Plus DNA Reagent Kit (Becton Dickinson). Flow cytometry was performed with a FACSort flow cytometer (Becton Dickinson), and the DNA histogram was analysed by a CELLQuest Version 1.2.2 (Becton Dickinson).

### Detection of apoptosis by flow cytometry

The ML-20 and KPL-4 cell lines were plated at a density of $4 \times 10^5$ cells per well in 6-well plates (SB Medical, Tokyo, Japan) and grown in DMEM supplemented with 5% FBS for 2 days. Then the cells were washed with PBS and treated with phenol red-free RPMI-1640 medium supplemented with 2% dextran-coated charcoal-stripped FBS plus $10^{-7}$ M ICI 182,780, 10 μg ml$^{-1}$ rhumAbHER2 or both. Duplicated wells were trypsinized and harvested 3 days after. The percentages of apoptotic cells were measured with a FACSCaliber flow cytometer (Becton Dickinson Immunocytometry Systems, Mansfield, MA, USA) using an in situ cell death detection kit (Boehringer Mannheim, Germany) according to the manufacturer's recommendations as described elsewhere (Otsuki et al, 1998).

### Detection of apoptosis by immunocytochemistry

The ML-20 and KPL-4 cell lines were plated at a density of $1 \times 10^6$ cells in T-75 flasks (Corning Japan, Tokyo, Japan) and grown in DMEM supplemented with 5% FBS for 3 days. Then the cells were washed with PBS and treated with phenol red-free RPMI-1640 medium supplemented with 2% dextran-coated charcoal-stripped FBS plus $10^{-7}$ M ICI 182,780, 10 μg ml$^{-1}$ rhumAbHER2 or both. Duplicated flasks were trypsinized and harvested 2 days after. The cell pellets were fixed with 5% buffered-formalin and embedded in paraffin. DNA breaks in the sections were detected by nick end labelling using an ApopTag in situ apoptosis detection kit (Oncor Inc., Gaithersburg, MD, USA) as described elsewhere (Kuwashima et al, 1996). The areas of most intense staining were identified in each sample, and approximately 500 cells per sample were examined. The percentage of cells showing positive staining was calculated in each sample.

### Statistical analysis

ER content, percentages of control cell numbers, percentages of the S phase or G0/G1 phase of the cell cycle and percentages of apoptotic cells were expressed as means ± s.e.m. These values for

*© 2000 Cancer Research Campaign* 

**Table 1** Comparison of expression levels of HER family members in three human breast cancer cell lines

| | ML-20 | KPL-4 | MDA-MB-231 |
|---|---|---|---|
| HER1 | 2 | 2 | 55 |
| HER2 | 14 | 13 | 5 |
| HER3 | 21 | 19 | 2 |
| HER4 | 4 | 1 | 1 |

The expression of each member was measured by flow cytometric analysis as described in the Materials and Methods. Each value represents the specific peak fluorescence intensity divided by the background peak intensity.

the control and treated groups were compared using analysis of variance (ANOVA) with StatView computer software (ATMS Co., Tokyo, Japan). All the experiments in this study were repeated at least twice and the reproducibility of the results was confirmed.

### RESULTS

### ER levels and HER family member expression

The ML-20 cells expressed a high level of ER (112 ± 9.9 fmol $mg^{-1}$ protein) but neither the KPL-4 cells nor the MDA-MB-231 cells expressed a detectable level of ER (the limit of detection of the ER assay was 5.0 fmol $mg^{-1}$ protein).

Flow cytometric analysis showed that both the ML-20 cells and the KPL-4 cells expressed a moderate level of HER2 and HER3 and a low level of HER1 and HER4. The MDA-MB-231 cells expressed a low level of HER2, HER3 and HER4 and a high level of HER1 (Table 1).

### Growth inhibitory effect of ICI 182,780, rhumAbHER2 or their combination

Both ICI 182,780 ($10^{-9}$–$10^{-7}$ M) alone and rhumAbHER2 (0.1–10 μg $ml^{-1}$) alone dose-dependently inhibited the growth of ML-20 cells (Figure 1 A, B). The inhibition rates were 0.40 for $10^{-7}$ M ICI 182,780 ($P$ = 0.0008 in comparison with the control) and 0.20 for 10 μg $ml^{-1}$ rhumAbHER2 ($P$ = 0.0094). rhumAbHER2 (0.1–10 μg $ml^{-1}$) also dose-dependently inhibited the growth of KPL-4 cells (Figure 1C). The inhibition rate was 0.14 for 10 μg $ml^{-1}$ rhumAbHER2 ($P$ = 0.0021). No growth inhibitory effect of ICI 182,780 was observed in either KPL-4 or MDA-MB-231 cells. rhumAbHER2 showed no effect on the MDA-MB-231 cell growth (data not shown).

To investigate a possible additive effect of rhumAbHER2 and ICI 182,780, 10 μg $ml^{-1}$ rhumAbHER2 and $10^{-7}$ M ICI 182,780 were concomitantly administered. The combined treatment inhibited the growth of ML-20 cells more than the respective agent alone (Figure 2A). The inhibition rates were 0.36 for $10^{-7}$ M ICI 182,780, 0.21 for 10 μg $ml^{-1}$ rhumAbHER2 and 0.55 for their combination. In contrast, no additive effect was observed in KPL-4 and MDA-MB-231 cells (Figure 2 B, C).

### Effects of ICI 182,780 and rhumAbHER2 on cell cycle progression and apoptosis

Both ICI 182,780 ($10^{-7}$ M) alone and rhumAbHER2 (10 μg $ml^{-1}$) alone significantly increased the percentage of the G0/G1 phase

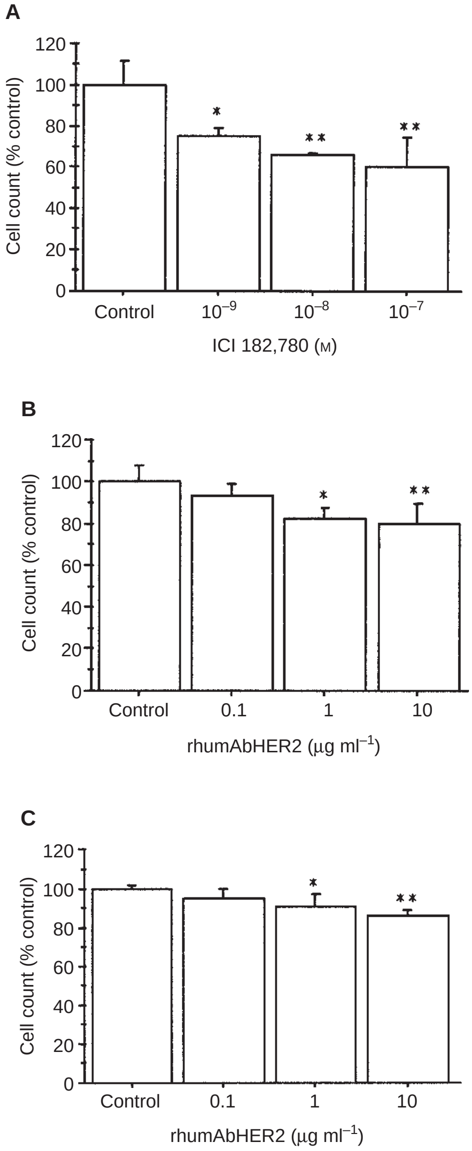

**Figure 1** Growth inhibitory effects of ICI 182,780 ($10^{-9}$–$10^{-7}$ M) in ML-20 cells (**A**), and those of rhumAbHER2 (0.1–10 μg $ml^{-1}$) in ML-20 cells (**B**) and in KPL-4 cells (**C**). The cells were incubated with medium containing the reagents for 4 days and counted with a Coulter counter. The values represent the percentages of the control and means of triplicate samples. Bars, SE. *$P$ < 0.05 in comparison with the control. **$P$ < 0.01 in comparison with the control

($P$ = 0.0001 and $P$ = 0.01 respectively, in comparison with the control) and decreased the percentage of S phase ($P$ = 0.0001 and $P$ = 0.0015 respectively) in ML-20 cells. In addition, the combined treatment promoted the G1–S blockade more than the respective agent alone (Table 2). In KPL-4 cells, on the other hand, rhumAbHER2 tended to increase the percentage of G0/G1 phase ($P$ = 0.39) and decrease the percentage of S phase ($P$ = 0.12), but no additive effect of the combined treatment was observed (Table 2).

Both ICI 182,780 ($10^{-7}$ M) alone and rhumAbHER2 (10 μg $ml^{-1}$) alone also induced apoptosis in the ML-20 cell line. In the flow cytometric analysis, ICI 182,780 alone or rhumAbHER2 alone tended to increase the percentage of TUNEL-positive cells but a

© 2000 Cancer Research Campaign

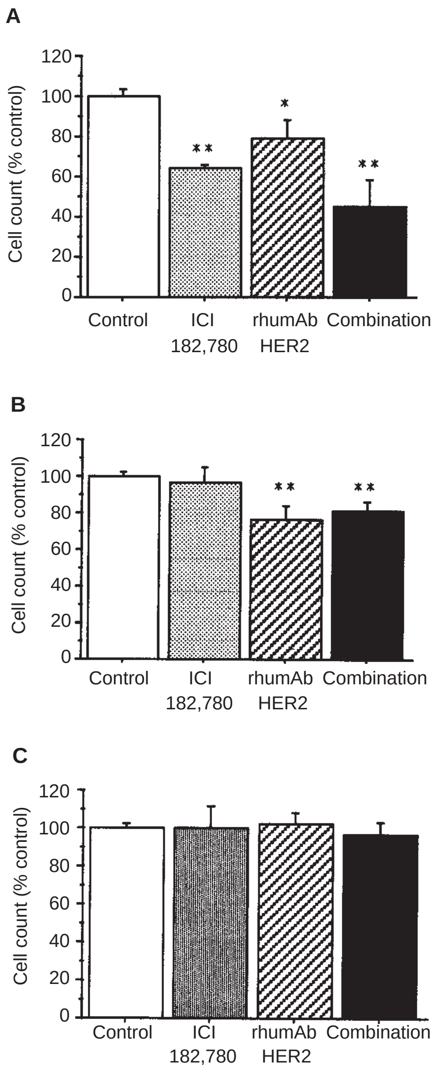

**Figure 2** Growth inhibitory effects of ICI 182,780 alone, 10 μg ml$^{-1}$ rhumAbHER2 alone or their combination in ML-20 cells (**A**), KPL-4 cells (**B**) or MDA-MB-231 cells (**C**). The cells were incubated with medium containing the reagents for 4 days and counted with a Coulter counter. The values represent the percentages of the control and means of triplicate samples. Bars, s.e.m. *$P$ < 0.05 in comparison with the control. **$P$ < 0.01 in comparison with the control

**Table 2** Effects of ICI 182,780 alone, rhumAbHER2 alone or their combination on cell cycle progression in ML-20 and KPL-4 human breast cancer cell lines

| Cell line | Treatment | % S | % G0/G1 |
|---|---|---|---|
| ML-20 | Control | 16.7 ± 0.3 | 75.6 ± 0.2 |
| | ICI 182,780 | 7.9 ± 1.1[b] | 85.3 ± 1.4[b] |
| | rhumAbHER2 | 13.7 ± 1.3[b] | 78.4 ± 0.7[a] |
| | Combination | 6.3 ± 0.4[b] | 86.6 ± 0.2[b] |
| KPL-4 | Control | 25.8 ± 0.5 | 60.9 ± 0.6 |
| | ICI 182,780 | 27.8 ± 1.2 | 59.2 ± 1.0 |
| | rhumAbHER2 | 23.8 ± 0.8 | 62.4 ± 1.4 |
| | Combination | 24.8 ± 1.3 | 62.1 ± 2.1 |

$10^{-7}$ M ICI 182,780 alone, 10 μg ml$^{-1}$ rhumAbHER2 alone or their combination was added to the culture medium and the cells were incubated for 3 days. Percentages of the S phase and G0/G1 of the cell cycle were measured by flow cytometric analysis as described in the Materials and Methods. The values represent means ± s.e.m. of triplicate samples. [a]$P$ < 0.05 in comparison with the control. [b]$P$ < 0.01 in comparison with the control.

**Table 3** Effects of ICI 182,780 alone, rhumAbHER2 alone or their combination on apoptosis measured by flow cytometric or immunocytochemical analysis in ML-20 and KPL-4 human breast cancer cell lines

| Assay method | Treatment | Cell line | |
|---|---|---|---|
| | | ML-20 | KPL-4 |
| Flow cytometry | Control | 4.9 ± 1.0 | 4.2 ± 0.7 |
| | ICI 182,780 | 8.7 ± 2.6 | 4.2 ± 0.2 |
| | rhumAbHER2 | 6.4 ± 0.0 | 4.9 ± 1.1 |
| | Combination | 10.5 ± 0.7[a] | 4.4 ± 0.9 |
| Immunocytochemistry | | | |
| | Control | 1.0 ± 0.5 | 2.7 ± 0.2 |
| | ICI 182,780 | 4.3 ± 0.8[b] | 2.9 ± 0.9 |
| | rhumAbHER2 | 2.9 ± 1.0[b] | 5.0 ± 0.4[b] |
| | Combination | 7.5 ± 1.5[b] | 4.7 ± 0.6[b] |

$10^{-7}$ M ICI 182,780 alone, 10 μg ml$^{-1}$ rhumAbHER2 alone or their combination was added to the culture medium and the cells were incubated for 2 days. Percentages of apoptotic cells were measured by flow cytometric or immunocytochemical analysis as described in the Materials and Methods. The values represent means ± s.e.m. of triplicate samples. [a]$P$ < 0.05 in comparison with the control. [b]$P$ < 0.01 in comparison with the control.

significant increase was noted with their combination ($P$ = 0.018 in comparison with the control, Table 3). In KPL-4 cells, rhumAbHER2 alone slightly increased the percentage of TUNEL-positive cells (Table 3). In the immunocytochemical analysis, both ICI 182,780 and rhumAbHER2 significantly induced apoptosis ($P$ = 0.0001 and $P$ = 0.0048 respectively) in ML-20 cells. The combined treatment also induced apoptosis more than the respective agent alone (Table 3). In KPL-4 cells, rhumAbHER2 alone significantly induced apoptosis ($P$ = 0.0001, Table 3), but no additive effect of the two agents on apoptosis was observed in KPL-4 cells.

A small difference was observed between the two apoptosis analyses in the percentages of apoptotic cells. The percentages in the flow cytometric analysis tended to be higher than those in the immunocytochemical analysis. These findings suggest the possibility that the immunocytochemical analysis is more specific in detecting apoptotic cells than the flow cytometric analysis.

### DISCUSSION

Antioestrogen resistance frequently occurs in hormone-dependent breast cancer following successful treatment with anti-oestrogen. It has been demonstrated that HER2-overexpressing MCF-7 cells which were transfected with a full-length of *HER2* cDNA were no longer sensitive to the anti-oestrogen tamoxifen (Benz el al, 1992). Thus, it has been speculated that HER2 overexpression may be one of the causes of anti-oestrogen resistance in human breast cancer.

Previous reports have suggested a direct interaction between ER and HER2-signalling pathways. It has been demonstrated that oestrogen down-regulates HER2 expression and anti-oestrogen partly reverses this oestrogen-induced HER2 down-regulation (Read et al, 1990). Overexpression of a ligand for HER3 or HER4, heregulin, which also stimulates the HER2-signalling pathway, down-regulates ER-mediated transcription (Tang et al, 1996). Our preliminary data on the regulation of HER2 expression by various

*© 2000 Cancer Research Campaign*

hormones in breast cancer cells also suggest that oestrogen down-regulates HER2 expression and anti-oestrogen up-regulates it (unpublished data). In addition, a recent clinical report suggested that an anti-oestrogen, tamoxifen, improves the outcome of patients with breast cancer without HER2-overexpression, while showing a paradoxical detrimental effect in patients with HER2-positive breast cancer (Bianco et al, 1998). These findings suggest that anti-oestrogen may promote breast cancer cell growth through the HER2-signalling pathway in ER- and HER2-positive breast cancer and prompted us to investigate a possible additive effect of anti-oestrogen and a blocker of HER2-signalling pathway, anti-HER2 antibody, in breast cancer cells.

It has been suggested that concomitant treatment with an anti-oestrogen, tamoxifen, and a murine monoclonal anti-HER2 antibody, 4D5, enhanced the anti-proliferative activity in ER- and HER2-positive BT474 breast cancer cells (Witters et al, 1997). In contrast, results of another experiment indicated that rhumAbHER2, which was used in the present study and a humanized form of 4D5, has no growth-inhibitory effect on MCF-7 cells (Lewis et al, 1993). The ML-20 cell line used in the present study is a transfectant of the MCF-7 cell line with a bacterial *lacZ* gene and its growth characteristics in vitro and in vivo are indistinguishable from the wild-type MCF-7 cell line (McLeskey et al, 1998). We confirmed that ML-20 cells express a high level of ER and a moderate level of HER2 (Table 1). rhumAbHER2 alone (0.1–10 $\mu g\ ml^{-1}$) dose-dependently inhibited the growth of ML-20 cells (Figure 1). The discrepancy in the effects of this antibody may be explained as follows. The phenol red-free medium supplemented with dextran-coated charcoal FBS used in the present study contains less endogenous oestrogen than the medium which was used in the other study. The lower level of oestrogen in our medium may induce HER2 expression in ML-20 cells and may enhance the growth inhibitory effect of rhumAbHER2.

To explore the mechanisms responsible for the growth inhibitory effect of ICI 182,780 and rhumAbHER2, their influences on cell cycle progression and apoptosis were investigated. It has been reported that anti-oestrogen causes a G1–S blockade and induces apoptosis in ER-positive breast cancer cells (Perry et al, 1995; Watts et al, 1995). It is also suggested that a murine anti-HER2 antibody induces apoptosis in HER2-overexpressing cancer cells (Kita et al, 1996). In the present study, ICI 182,780 significantly induced a G1–S blockade and apoptosis in ER-positive ML-20 cells but not in ER-negative KPL-4 cells. rhumAbHER2 also significantly induced a G1–S blockade and apoptosis in ML-20 cells, which express a moderate level of HER2, but not in MDA-MB-231 cells, which express a low level of HER2. In KPL-4 cells, rhumAbHER2 caused a significant induction of apoptosis and a slight G1–S blockade. Interestingly, the combined treatment with ICI 182,780 and rhumAbHER2 enhanced the induction of either a G1–S blockade or apoptosis in ML-20 cells which express both ER and HER2 (Tables 2 and 3). These findings in the present study support the findings in previous reports indicating that either ICI 182,780 alone or rhumAbHER2 alone causes a G1–S blockade and apoptosis in breast cancer cells and this results in the retardation of cell growth.

Combined treatment with rhumAbHER2 and a certain cytotoxic agent, such as cisplatin, paclitaxel or doxorubicin/cyclophosphamide, has been reported to be a promising approach for the treatment of advanced breast cancer (Pegram et al, 1998; Slamon et al, 1998). However, the action mechanisms responsible for their additive effect remain to be elucidated. In addition, an unexpected adverse effect of their combination; that is, cardiotoxicity, has been reported (Slamon et al, 1998). rhumAbHER2 may also enhance the anti-tumour effect of hormonal agents, such as anti-oestrogen, as indicated in this study. Thus, it is conceivable that combined treatment with a HER2-signalling blocker and a hormonal agent might be a new promising therapeutic approach for ER- and HER2-positive breast cancer.

### ACKNOWLEDGEMENTS

This work is partly supported by grants from the Ministry of Education, Science, Sports and Culture of Japan (to J Kurebayashi), from the Ministry of Health of Japan (to H Sonoo) and by Research Grants (no. 10–113 to J Kurebayashi and no. 10–307 to H Sonoo) from Kawasaki Medical School.

### REFERENCES

Baselga J, Tripathy D, Mendelsohn J, Baughman S, Benz CC, Dantis L, Skalarin NT, Seidman AD, Hudis CA, Moore J, Rosen PP, Twaddell T, Henderson IC and Norton L (1996) Phase II study of weekly intravenous recombinant humanized anti-p185$^{HER2}$ monoclonal antibody in patients with HER2/*neu*-overexpressing metastatic breast cancer. *J Clin Oncol* **14**: 737–744

Baselga J, Norton L, Albanell J, Kim YM and Mendelsohn J (1998) Recombinant humanized anti-HER2 antibody (Herceptin) enhances the antitumor activity of paclitaxel and doxorubicin against HER2/neu overexpressing human breast cancer xenografts. *Cancer Res* **58**: 2825–2831

Benz CC, Scott GK, Sarup JC, Johnson RM, Tripathy D, Coronado E, Shepard HM and Osborne CK (1992) Estrogen-dependent, tamoxifen-resistant tumorigenic growth of MCF-7 cells transfected with HER2/neu. *Breast Cancer Res Treat* **24**: 85–95

Bianco AR, Laurentis MDe, Carlomgno C, Lauria R, Petrella G, Pianco L, Pettinato G, Perrone F, Gallo C, Marinelli A and De Placido S (1998) Twenty-year update the Naples gun trial of adjuvant breast cancer therapy: evidence of interaction between c-erbB2 expression and tamoxifen efficacy. *Proc ASCO* **17**: 97

Carter P, Presta L, Gorman CM, Ridway JBB, Henner D, Wong WLT, Rowland AM, Kotts C, Carver ME and Shepard HM (1992) Humanization of an anti-p 185HER2 antibody for human cancer therapy. *Proc Natl Acad Sci USA* **89**: 4285–4289

Cobleigh MA, Vogel CL, Tripathy D, Robert NJ, Scholl S, Fehrenbacher L, Paton V, Shak S, Lieberman G and Slamon D (1998) Efficacy and safety of HERCEPTIN (humanized anti-HER2 antibody) as a single agent in 222 women with HER2 overexpression who relapsed following chemotherapy. *Proc ASCO* **17**: 97

Coussens L, Yang-Feng TL, Liao YC, Chen E, Gray A, McGrath J, Seeberg PhH, Lebermann TA, Schlessinger J, Franke U, Levinson A and Ullrich A (1985) Tyrosine kinase receptor with extensive homology to EGF receptor shares chromosomal location with the neu oncogene. *Science* **230**: 1130–1139

Fukushige SI, Matsubara K and Yoshida M (1986) Localisation of a novel verbB-related gene, c-erbB-2, on chromosome-17 and its amplification in a gastric cell line. *Mol Cell Biol* **6**: 955–958

Kita Y, Tseng J, Horan T, Wen J, Philo J, Chang D Ratzkin B, Pacifici R, Branknow D, Hu S, Luo Y, Wen D, Arakawa T and Nicolson M (1996) ErbB receptor activation, cell morphology changes, and apoptosis induced by anti-Her2 monoclonal antibodies. *Biochem Biophys Res Commun* **226**: 59–69

Kurebayashi J, Otsuki T, Yamamoto S, Kurosumi M, Nakata T, Akinaga S and Sonoo H (1998) A pure antioestrogen, ICI 182,780, stimulates the growth of tamoxifen-resistant KPL-1 human breast cancer cells in vivo but not in vitro. *Oncology* **55S**: 23–34

Kurebayashi J, Tang CK, Otsuki T, Kurosumi M, Yamamoto S, Tanaka K, Mochizuki M, Nakamura H and Sonoo H (1999) Isolation and characterisation of a new human breast cancer cell line, KPL-4, expressing Erb B family receptors and interleukin-6. *Br J Cancer* **79**: 707–717

Kuwashima Y, Kobayashi Y, Kawarai A, Uehara T, Kurosumi M, Tanuma J, Shiromizu K, Katsuzawa M and Kishi K (1996) Expression of bcl-2 and apoptotic DNA fragmentation in human endoterial adenocarcinoma cells. *Anticancer Res* **16**: 3221–3224

Lewis GD, Figari I, Fendly B, Wong WL, Carter P, Gorman C and Shepard HM (1993) Differential responses of human tumor cell lines to anti-p 185HER2 monoclonal antibodies. *Cancer Immunol Immunother* **37**: 255–263

 © 2000 Cancer Research Campaign

McCann AH, Dervan PA, O'Regan M, Codd MB, Gullick WJ, Tobin BMJ and Carney DN (1991) Prognostic significance of c-erbB-2 and estrogen receptor status in human breast cancer. *Cancer Res* **50**: 3296–3303

McLeskey SW, Zhang L, El-Ashry D, Trock BJ, Lopez CA, Kharbanda S, Tobios CA, Lorant LA, Hunnum RS, Dickson RB and Kern FG (1998) Tamoxifen-resistant fibroblast growth factor-transfected MCF-7 cells are cross-resistant in vivo to the antiestrogen ICI 182,780 and two aromatase inhibitors. *Clin Cancer Res* **4**: 497–711

Maguire HC and Greene MI (1989) The neu (c-erbB-2) oncogene. *Semin Oncol* **16**: 148–155

Otsuki T, Yamada O, Sakaguchi H, Tomokuni A, Wada H, Yawata Y and Ueki A (1998) Human myeloma cell apoptosis induced by interferon-alpha. *Br J Haematol* **103**: 518–529

Pegram MD, Lipton A, Hayes DF, Weber BL, Baselga JM, Tripathy D, Baly D, Baughman SA, Twaddell T, Glaspy JA and Slamon DJ (1998) Phase II study of receptor-enhanced chemosensitivity using recombinant humanized anti-p185HER2/neu monoclonal antibody plus cisplatin in patients with HER2/neu-overexpressing metastatic breast cancer refractory to chemotherapy treatment. *J Clin Oncol* **16**: 2659–2671

Perry RR, King Y and Greaves BR (1995) Relationship between tamoxifen-induced transforming growth factor β1 expression, cytostasis and apoptosis in human breast cancer cells. *Br J Cancer* **72**: 1441–1446

Read LD, Keith D, Slamon DJ and Katzenellenbogen BS (1990) Hormonal modulation of HER2/neu protooncogene messenger ribonucleic acid and p185 protein expression in human breast cancer cell lines. *Cancer Res* **50**: 3947–3951

Ross JS and Fletcher JA (1998) The HER/neu oncogene in breast cancer: prognostic factor, predictive factor, and target for therapy. *Stem Cell* **16**: 413–428

Schecter AL, Stern DF, Vaidyanathan L and Decker SJ (1984) The neu oncogene: an erbB-related gene encoding a 185,000 Mr tumour antigen. *Nature* **312**: 513–516

Slamon DJ, Clark GM, Wong SG, Levin WJ, Ullrich A and McGuire WL (1987) Human breast cancer: correlation of relapse and survival with amplification of the HER2/neu oncogene. *Science* **235**: 177–182

Slamon D, Layland-Jones B, Shak S, Paton V, Bajamonde A, Fleming T, Eiemann W, Wolter J and Norton L (1998) Addition of HERCEPTIN (humanized anti-HER2 antibody) to first line chemotherapy for HER2 overexpressing metastatic breast cancer (HER2+/MBC) markedly increases anticancer activity: a randomized, multinational controlled phase II trial. *Proc ASCO* **17**: 98

Tang CK, Perez C, Grunt T, Waibel C, Cho C and Lupu R (1996) Involvement of heregulin-β2 in the acquisition of the hormone-independent phenotype of breast cancer cells. *Cancer Res* **56**: 3350–3358

Tokuda Y, Ohnishi Y, Shimamura K, Iwasawa M, Yoshimura M, Ueyama Y, Tamaoki N, Tajima T and Mitomi T (1996) In vitro and in vivo anti-tumour effects of a humanised monoclonal antibody against c-erbB-2 product. *Br J Cancer* **73**: 1362–1365

Watts CK, Brady A, Sarcevic B, deFazio A, Musgrove EA and Sutherland RL (1995) Antiestrogen inhibition of cell cycle progression in breast cancer cells in association with inhibition of cyclin-dependent kinase activity and decreased retinoblastoma protein phosphorylation. *Mol Endocrinol* **9**: 1804–1813

Witters LM, Kumar R, Chinchilli VM and Lipton A (1997) Enhanced anti-proliferative activity of the combination of tamoxifen plus HER-2-neu antibody. *Breast Cancer Res Treat* **42**: 1–5

Wright C, Angus B, Nicholson S, Sainsbury JRC, Cairns J, Gullick WJ, Kelly P, Harris AL and Wilson Horne CH (1989) Expression of c-erbB-2 oncoprotein: a prognostic indicator in human breast cancer. *Cancer Res* **49**: 2087–2090

*© 2000 Cancer Research Campaign*

## References

[PDF_00557] Baselga J., Norton L., Albanell J., Kim Y. M., Mendelsohn J. (1998). Recombinant humanized anti-HER2 antibody (Herceptin) enhances the antitumor activity of paclitaxel and doxorubicin against HER2/neu overexpressing human breast cancer xenografts.. Cancer Res.

[PDF_00551] Baselga J., Tripathy D., Mendelsohn J., Baughman S., Benz C. C., Dantis L., Sklarin N. T., Seidman A. D., Hudis C. A., Moore J. (1996). Phase II study of weekly intravenous recombinant humanized anti-p185HER2 monoclonal antibody in patients with HER2/neu-overexpressing metastatic breast cancer.. J Clin Oncol.

[PDF_00561] Benz C. C., Scott G. K., Sarup J. C., Johnson R. M., Tripathy D., Coronado E., Shepard H. M., Osborne C. K. (1992). Estrogen-dependent, tamoxifen-resistant tumorigenic growth of MCF-7 cells transfected with HER2/neu.. Breast Cancer Res Treat.

[PDF_00570] Carter P., Presta L., Gorman C. M., Ridgway J. B., Henner D., Wong W. L., Rowland A. M., Kotts C., Carver M. E., Shepard H. M. (1992). Humanization of an anti-p185HER2 antibody for human cancer therapy.. Proc Natl Acad Sci U S A.

[PDF_00580] Coussens L., Yang-Feng T. L., Liao Y. C., Chen E., Gray A., McGrath J., Seeburg P. H., Libermann T. A., Schlessinger J., Francke U. (1985). Tyrosine kinase receptor with extensive homology to EGF receptor shares chromosomal location with neu oncogene.. Science.

[PDF_00583] Fukushige S., Matsubara K., Yoshida M., Sasaki M., Suzuki T., Semba K., Toyoshima K., Yamamoto T. (1986). Localization of a novel v-erbB-related gene, c-erbB-2, on human chromosome 17 and its amplification in a gastric cancer cell line.. Mol Cell Biol.

[PDF_00586] Kita Y., Tseng J., Horan T., Wen J., Philo J., Chang D., Ratzkin B., Pacifici R., Brankow D., Hu S. (1996). ErbB receptor activation, cell morphology changes, and apoptosis induced by anti-Her2 monoclonal antibodies.. Biochem Biophys Res Commun.

[PDF_00594] Kurebayashi J., Otsuki T., Tang C. K., Kurosumi M., Yamamoto S., Tanaka K., Mochizuki M., Nakamura H., Sonoo H. (1999). Isolation and characterization of a new human breast cancer cell line, KPL-4, expressing the Erb B family receptors and interleukin-6.. Br J Cancer.

[PDF_00590] Kurebayashi J., Otsuki T., Yamamoto S., Kurosumi M., Nakata T., Akinaga S., Sonoo H. (1998). A pure antiestrogen, ICI 182,780, stimulates the growth of tamoxifen-resistant KPL-1 human breast cancer cells in vivo but not in vitro.. Oncology.

[PDF_00598] Kuwashima Y., Kobayashi Y., Kawarai A., Uehara T., Kurosumi M., Tanuma J., Shiromizu K., Matsuzawa M., Kishi K. (1996). Expression of bcl-2 and apoptotic DNA fragmentation in human endometrial adenocarcinoma cells.. Anticancer Res.

[PDF_00602] Lewis G. D., Figari I., Fendly B., Wong W. L., Carter P., Gorman C., Shepard H. M. (1993). Differential responses of human tumor cell lines to anti-p185HER2 monoclonal antibodies.. Cancer Immunol Immunother.

[PDF_00615] Maguire H. C., Greene M. I. (1989). The neu (c-erbB-2) oncogene.. Semin Oncol.

[PDF_00607] McCann A. H., Dervan P. A., O'Regan M., Codd M. B., Gullick W. J., Tobin B. M., Carney D. N. (1991). Prognostic significance of c-erbB-2 and estrogen receptor status in human breast cancer.. Cancer Res.

[PDF_00610] McLeskey S. W., Zhang L., El-Ashry D., Trock B. J., Lopez C. A., Kharbanda S., Tobias C. A., Lorant L. A., Hannum R. S., Dickson R. B. (1998). Tamoxifen-resistant fibroblast growth factor-transfected MCF-7 cells are cross-resistant in vivo to the antiestrogen ICI 182,780 and two aromatase inhibitors.. Clin Cancer Res.

[PDF_00617] Otsuki T., Yamada O., Sakaguchi H., Tomokuni A., Wada H., Yawata Y., Ueki A. (1998). Human myeloma cell apoptosis induced by interferon-alpha.. Br J Haematol.

[PDF_00620] Pegram M. D., Lipton A., Hayes D. F., Weber B. L., Baselga J. M., Tripathy D., Baly D., Baughman S. A., Twaddell T., Glaspy J. A. (1998). Phase II study of receptor-enhanced chemosensitivity using recombinant humanized anti-p185HER2/neu monoclonal antibody plus cisplatin in patients with HER2/neu-overexpressing metastatic breast cancer refractory to chemotherapy treatment.. J Clin Oncol.

[PDF_00626] Perry R. R., Kang Y., Greaves B. R. (1995). Relationship between tamoxifen-induced transforming growth factor beta 1 expression, cytostasis and apoptosis in human breast cancer cells.. Br J Cancer.

[PDF_00629] Read L. D., Keith D., Slamon D. J., Katzenellenbogen B. S. (1990). Hormonal modulation of HER-2/neu protooncogene messenger ribonucleic acid and p185 protein expression in human breast cancer cell lines.. Cancer Res.

[PDF_00633] Ross J. S., Fletcher J. A. (1998). The HER-2/neu oncogene in breast cancer: prognostic factor, predictive factor, and target for therapy.. Stem Cells.

[PDF_00635] Schechter A. L., Stern D. F., Vaidyanathan L., Decker S. J., Drebin J. A., Greene M. I., Weinberg R. A. (1984). The neu oncogene: an erb-B-related gene encoding a 185,000-Mr tumour antigen.. Nature.

[PDF_00638] Slamon D. J., Clark G. M., Wong S. G., Levin W. J., Ullrich A., McGuire W. L. (1987). Human breast cancer: correlation of relapse and survival with amplification of the HER-2/neu oncogene.. Science.

[PDF_00646] Tang C. K., Perez C., Grunt T., Waibel C., Cho C., Lupu R. (1996). Involvement of heregulin-beta2 in the acquisition of the hormone-independent phenotype of breast cancer cells.. Cancer Res.

[PDF_00649] Tokuda Y., Ohnishi Y., Shimamura K., Iwasawa M., Yoshimura M., Ueyama Y., Tamaoki N., Tajima T., Mitomi T. (1996). In vitro and in vivo anti-tumour effects of a humanised monoclonal antibody against c-erbB-2 product.. Br J Cancer.

[PDF_00653] Watts C. K., Brady A., Sarcevic B., deFazio A., Musgrove E. A., Sutherland R. L. (1995). Antiestrogen inhibition of cell cycle progression in breast cancer cells in associated with inhibition of cyclin-dependent kinase activity and decreased retinoblastoma protein phosphorylation.. Mol Endocrinol.

[PDF_00657] Witters L. M., Kumar R., Chinchilli V. M., Lipton A. (1997). Enhanced anti-proliferative activity of the combination of tamoxifen plus HER-2-neu antibody.. Breast Cancer Res Treat.

[PDF_00660] Wright C., Angus B., Nicholson S., Sainsbury J. R., Cairns J., Gullick W. J., Kelly P., Harris A. L., Horne C. H. (1989). Expression of c-erbB-2 oncoprotein: a prognostic indicator in human breast cancer.. Cancer Res.

